# Using a Brief Body Sway Assessment Device to Track Balance Differences across the Huntington's Disease Integrated Staging System Spectrum

**DOI:** 10.1002/mdc3.70288

**Published:** 2025-09-08

**Authors:** Japleen Kaur, Nadeen Youhanan, Krisha Bagga, Andrew Hall, Paul E. Gilbert, Daniel J. Goble, Jody Corey‐Bloom

**Affiliations:** ^1^ Department of neuroscience UC San Diego San Diego California USA; ^2^ Department of Psychology San Diego State University San Diego California USA; ^3^ Exercise Science, Oakland University Rochester Michigan USA

**Keywords:** Balance, falls, HD‐ISS staging system, Huntington's Disease

## Abstract

**Background:**

Huntington's disease (HD) is an autosomal dominant neurodegenerative disorder caused by a mutation in the *huntingtin* gene on chromosome 4, leading to progressive cognitive decline, motor impairment, and functional disability. Although balance impairment is recognized in HD, its onset and evolution with disease stage remain poorly understood.

**Objective:**

The aim was to track the onset and evolution of balance impairment in HD with progression of disease stage using the BTrackS Balance Plate.

**Methods:**

Total body sway (TBS) was assessed in 123 gene‐positive participants and 33 healthy controls (HC) using the BTrackS Balance Plate and laptop software. The prognostic index–derived Huntington's Disease Integrated Staging System (HD‐ISS) was used to stratify these subjects into stage 0/1 (n = 51), stage 2 (n = 38), and Stage 3 (n = 34). Nonparametric receiver operating characteristic curve analysis was used to compute optimal cutoff values for TBS.

**Results:**

Balance assessment revealed significant differences in TBS between HCs and gene‐positive participants (*P* < 0.001). TBS varied significantly across disease stages (*P* < 0.001), with mean values of 9.56 cm (stage 0/1), 14.46 cm (stage 2), and 28.26 cm (Stage 3). The comparison between HCs and stage 0/1 individuals revealed strong discrimination (area under the curve [AUC] = 0.709), with a threshold of 7.72 cm achieving 74.5% sensitivity. The most robust discrimination emerged between stage 2 and Stage 3 participants (AUC = 0.71), with a threshold of 11.85 cm at 82.4% sensitivity.

**Conclusions:**

In conclusion, this cross‐sectional study demonstrates that balance impairment is an early and progressive feature of HD, detectable even before the onset of overt motor symptoms.

Huntington's disease (HD) is an autosomal dominant neurodegenerative disorder caused by a mutation in the *huntingtin* gene on chromosome 4, leading to progressive cognitive decline, motor impairment, and functional disability.^1^ The basal ganglia and its associated neural circuits are crucial for maintaining postural control and regulating muscle tone.^2^ Early degeneration within these structures contributes to gait disturbances and postural instability, ultimately impacting balance.^3^, ^4^ Indeed, gait and balance dysfunction are strong predictors of diminished quality of life in individuals with HD.^5^ Currently, the clinical diagnosis of HD is based only on the diagnostic confidence level from the United Huntington's Disease Rating Scale (UHDRS) motor exam in the context of a confirmed gene status.^6^

Although balance impairment is recognized in HD,^5^, ^7^, ^8^, ^9^, ^10^ its onset and evolution with disease stage remain poorly understood. Some studies have suggested that balance problems may begin during the pre‐manifest stage, even before a clinical diagnosis is established.^8^ Various tools have been used to quantify balance disturbance in HD, including the Wii balance board,^7^ Tinetti Mobility Test, Timed Up and Go (TUG) test, chair sit‐to‐stand (CST), and the Berg Balance Scale.^9^, ^10^, ^11^ Recently, dynamic posturography has been proposed as a useful tool to find subtle changes in postural stability in HD.^12^ However, identifying meaningful differences between pre‐manifest individuals and healthy controls (HC) has been challenging.^12^ To the best of our knowledge, this is the first study to systematically investigate how balance impairment changes across the spectrum of HD severity, as measured using the HD‐ISS.

The primary challenge lies in the inadequacy of traditional staging methods to characterize disease progression. The UHDRS Total Motor Score (TMS), which assesses motor function, and total functional capacity (TFC), which measures functional independence,^13^ suffer from significant reliability issues.^12^, ^14^ It has been suggested that subtle motor abnormalities are poor markers of HD phenoconversion, with low interrater reliability, leading to symptoms that are missed or misinterpreted.^15^ This makes it difficult to accurately assess and compare balance impairment in different phases of HD using traditional staging methods in a cross‐sectional design.

To address these critical gaps, our study utilizes the Huntington's Disease Integrated Staging System (HD‐ISS) developed by Tabrizi et al.^16^ to examine balance impairment across different disease stages in a cross‐sectional sample of individuals with HD. Unlike traditional staging, which relies primarily on clinical observations,^13^ HD‐ISS incorporates biomarkers and imaging data to provide a more comprehensive and biologically driven assessment of disease progression. Stage 1 of the HD‐ISS captures the early neurodegeneration characterized by caudate and/or putamen atrophy, occurring before clear motor symptoms (stage 2) and functional decline (Stage 3). We hypothesized that these early structural changes may manifest as detectable alterations in postural stability. To assess this, we employed the BTrackS Balance Plate,^17^ which uses total body sway (TBS) and center of pressure (COP) technology to provide precise measurements of postural stability. Due to the link between motor abnormalities and functional decline, early identification and characterization of balance deficits are necessary for timely intervention.^12^, ^18^

## Patients and Methods

### Ethics Statement

This cross‐sectional study was conducted at the University of California, San Diego (UCSD) Huntington's Disease Society of America Centre of Excellence (HDSA CoE), with study approval from the UCSD Institutional Review Board (IRB) Committee (IRB protocol no. 170038) and in accordance with the requirements of the Code of Federal Regulations on the Protection of Human Subjects.

### Participants

We enrolled 123 HD individuals and 33 HCs. HD patients, confirmed by expanded CAG repeats, and HCs were recruited from the Huntington's Disease Center of Excellence at the University of California, San Diego. Individuals over 65 years of age, or with a history of musculoskeletal injury, orthopedic surgery, or peripheral neuropathy, were excluded. All study procedures were approved by the IRB, and informed consent was obtained from each participant before data collection.

### Assessments

The UHDRS was administered to all participants. This included the TFC, TMS, and Total Maximal Chorea score. Cognitive assessments included the Symbol Digit Modality Test (SDMT), Stroop Word Reading (SWR) test, Verbal Fluency Test, and Mini‐Mental State Examination (MMSE). The composite UHDRS (cUHDRS) score^19^ was calculated for each participant. Balance was assessed using the TUG, CST, and BTrackS Balance Assessment. Body mass index (BMI) was also calculated for all participants due to its potential influence on COP.^20^

### BTrackS Balance Plate and Data Collection

The BTrackS Balance Plate^17^ is a Food and Drug Administration–registered force plate that measures COP during standing. It is lightweight (<7 kg) with a surface area of 0.4 × 0.6 m and uses 4‐sensor technology with strain gauge load sensors and sampling data at 25 Hz. Sensors beneath each foot enable precise balance detection, with each sensor wired to a bridge‐type circuit board providing force‐related voltage signals. Force plates under the participant's feet are capable of quantifying balance by tracking changes in the individual's COP and total body movement. Custom software integrates these factors, yielding TBS through spatially weighted averages across different conditions. TBS represents the cumulative distance traveled by the COP during the measurement period, with higher values indicating greater postural instability. The BTrackS system has demonstrated strong concurrent validity with research‐grade force platforms and excellent test–retest reliability.^21^

The BTrackS Balance Plate was placed on a firm, level surface and leveled using adjustable legs and a bubble leveling tool per the manufacturer's specifications. Data were collected using the BTrackS Sport Balance software on an ASUS laptop (model X200) with a Windows 8.1 operating system connected to the balance plate via a USB cable.

To measure TBS, participants stood on the balance plate with their eyes open, hands on their hips, feet shoulder‐width apart, and heads straight. Four 10‐s trials were conducted for each participant, and the average TBS (in centimeters of deviation from the starting COP) was calculated.

### Huntington's Disease Integrated Staging System

Participants were stratified into stages based on their normalized prognostic index (PIN) score^22^ using the HD‐ISS.^16^ Stage 0 includes gene mutation carriers (CAG > 40). Stage 1 represents participants with caudate and/or putamen atrophy before onset of clinical symptoms. In the absence of any clinical symptoms in stages 0 and 1, for this analysis, we chose to combine the 2 stages, as suggested by Long et al.^23^ Stage 2 includes participants who are HD gene carriers and have motor and/or cognitive decline but are functionally not impacted; Stage 3 participants are HD gene carriers who have functional decline as a result of their disease progression.

### Statistical Analysis

Data were analyzed using GraphPad Prism 9.5.1 and SPSS Statistics for Macintosh. A Shapiro–Wilk test revealed that the data were not normally distributed (*P* < 0.01), so nonparametric analyses were used. A Kruskal–Wallis test with Bonferroni correction was used to compare performance among cohorts, and Dunn's multiple comparisons test was used for post hoc analyses. Receiver operating characteristic (ROC) curve analysis was conducted to identify optimal cutoff values for TBS in differentiating between groups (HC vs. gene positive, HC vs. stage 0/1, and stage 2 vs. Stage 3).

## Results

### Demographics and Clinical Characteristics

The study included data from 33 HCs and 123 gene‐positive participants distributed across HD‐ISS stages: 51 in stage 0/1, 38 in stage 2, and 34 in Stage 3, representing disease progression from early to advanced states. Mean age increased significantly with disease stage (*P* < 0.001), from 39.45 years in stage 0/1 to 50.92 in stage 2 to 49.85 in Stage 3. BMI did not vary significantly among cohorts (*P* > 0.05) (Table 1). Cognitive function declined significantly with disease progression, as evidenced by decreasing scores in MMSE, Montreal Cognitive Assessment, SDMT, and SWR test (*P* < 0.001). Although motor impairment was minimal in the early stages (mean TMS score = 0.49 in stage 0/1), it worsened significantly in later stages (mean TMS score = 13.70 in stage 2 and 30.09 in Stage 3; *P* < 0.001). Chorea scores also increased significantly, starting at a mean of 0.49 in stage 0/1, increasing to 3.97 in stage 2, and reaching 5.5 in Stage 3 (*P* < 0.001).

**TABLE 1:** Demographics and clinical characteristics for HCs and gene‐positive participants stratified by the HD‐ISS, mean (standard deviation)

	Healthy controls	Stage 0/1	Stage 2	Stage 3	*P*‐value
n	33	51	38	34	–
Age (yr)	46.45 (12.83)	39.45 (11.21)	50.92 (10.03)	49.85 (8.49)	<0.001
Gender (M/F)	14/19	23/28	20/18	17/17	0.81
Education (yr)	15.5 (3.52)	15.47 (2.55)	15.70 (2.77)	14.50 (2.86)	0.264
BMI	26.01 (5.81)	25.11 (4.71)	25.41 (3.57)	23.68 (4.72)	0.13
MMSE	29.30 (0.76)	27.90 (2.25)	27.16 (2.12)	25.12 (3.17)	<0.001
SDMT	57.84 (10.41)	53.43 (9.61)	36.89 (9.12)	25.47 (9.03)	<0.001
TFC	13 (0)	41.94 (12.53)	11.53 (1.71)	9.76 (2.52)	<0.001
SWR	100.12 (13.67)	94.54 (19.17)	82.68 (19.35)	59.03 (16.85)	<0.001
TMS	0.06 (0.35)	3.33 (5.42)	13.70 (8.36)	30.09 (12.45)	<0.001
TMC	0 (0)	0.49 (0.98)	3.97 (2.85)	5.5 (3.63)	<0.001
TUG (s)	8.65 (1.31)	9.25 (1.75)	9.94 (2.57)	10.35 (1.91)	0.006
CST	14.39 (4.17)	11.66 (3.72)	14.34 (5.08)	12.31 (3.84)	0.02
TBS (cm)	7.51 (2.64)^a^	9.56 (2.96)^a^, ^b^	14.46 (8.28)^a^, ^b^, ^c^	28.26 (30.69)^a^, ^c^	<0.001
cUHDRS	18.09 (1.20)	16.86 (1.81)	14.16 (1.92)	10.66 (2.40)	<0.001

Cohort performance compared using nonparametric Kruskal–Wallis test corrected with Bonferroni; multiple comparisons performed using Dunn's multiple comparisons.

Abbreviations: HC, healthy control; HD‐ISS, Huntington's Disease Integrated Staging System; BMI, body mass index; MMSE, Mini‐Mental State Examination; SDMT, Symbol Digit Modality Test; TFC, total functional capacity; SWR, Stroop Word Reading test; TMS, Total Motor Score; TMC, Total Maximal Chorea score; TUG, Timed Up and Go test; CST, chair sit‐to‐stand; TBS, total body sway; cUHDRS, composite United Huntington's Disease Rating Scale.

^a^ Significant differences between HCs and all gene‐positive participants, *P* < 0.001.

^b^ Significant differences between stages 0/1 and 2, *P* < 0.001.

^c^ Significant differences between stages 2 and 3, *P* = 0.027.

### Balance Assessment

Balance assessment revealed significant differences in TBS between HCs and gene‐positive participants (*P* < 0.001; Table 1). TBS was significantly higher in stage 0/1 participants (9.56 cm) compared to HCs (7.51 cm, *P* = 0.02), suggesting that balance impairment may begin early in HD. TBS progressively increased across disease stages, with mean values of 9.56 cm (stage 0/1), 14.46 cm (stage 2), and 28.26 cm (Stage 3), with significant variation between stages (*P* < 0.001). Pairwise comparisons showed significant differences between stages 0/1 and 2 (*P* < 0.001) and between stages 2 and 3 (*P* = 0.02).

**FIG. 1:**
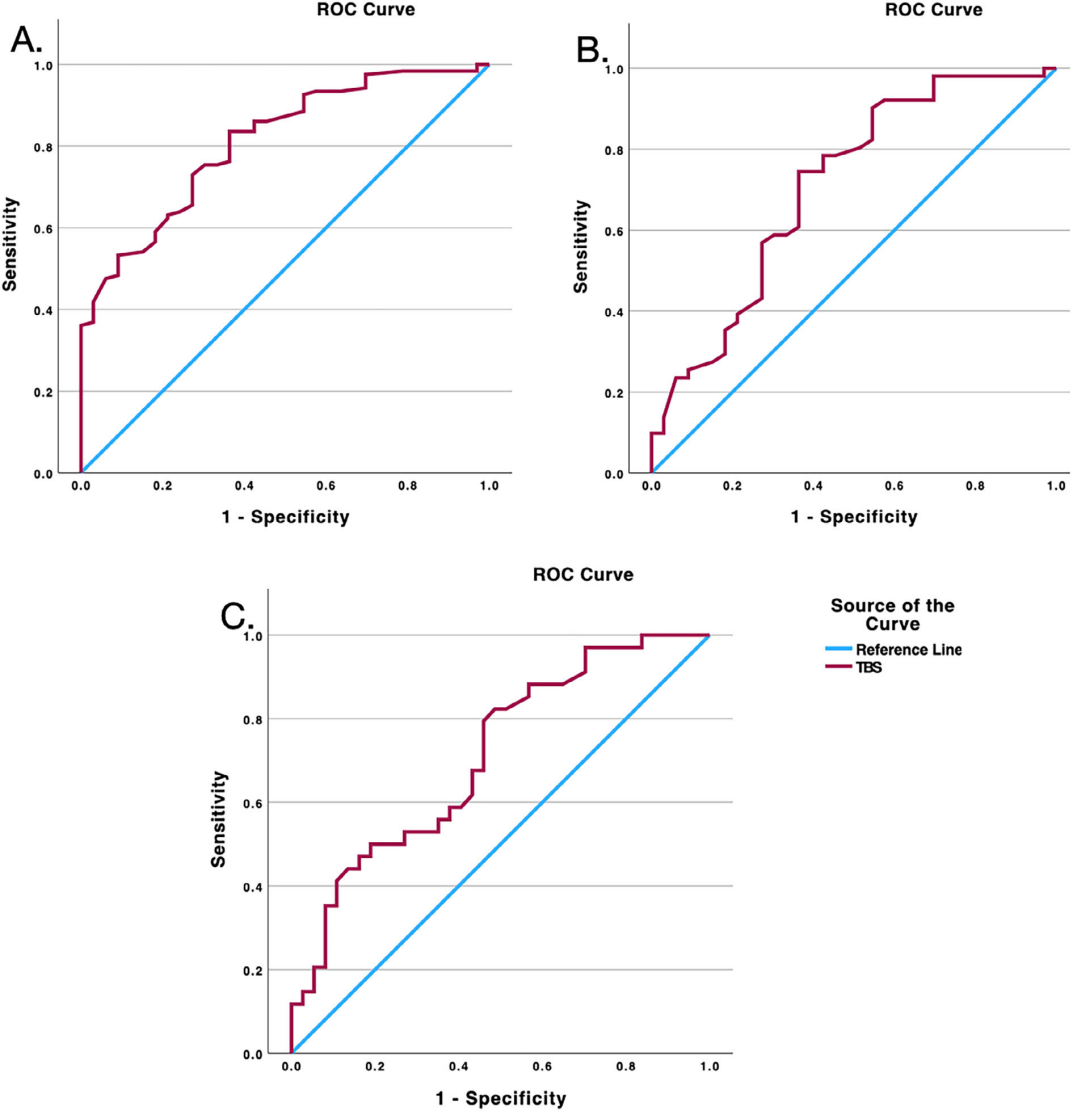
Receiver operator characteristic (ROC) curve showing TBS (total body sway) between (**A**) HCs (healthy controls) and all gene‐positive subjects: area under the curve (AUC): 0.81, threshold: 7.72 cm, sensitivity: 83.6%, and specificity: 68.2%; (**B**) HCs and stage 01/1 participants: AUC: 0.709, threshold: 7.72 cm, sensitivity: 74.5%, and specificity: 61.8%; (**C**) stage 2 and Stage 3 participants: AUC: 0.71, threshold: 11.85 cm, sensitivity: 82.4%, and specificity, 51.4%.

Our ROC analyses (Fig. 1) examined 3 key comparisons to assess the discriminatory ability of TBS measurements. The analysis between HCs and gene‐positive individuals showed strong discriminatory ability (area under the curve [AUC] = 0.788), with an optimal threshold of 7.72 cm achieving 83.6% sensitivity (Fig. 1A). The comparison between HCs and stage 0/1 individuals revealed similar discrimination (AUC = 0.709), with a threshold of 7.72 cm achieving 74.5% sensitivity (Fig. 1B). The most robust discrimination emerged between stage 2 and Stage 3 participants. Using a threshold of 11.85 cm, the analysis achieved 82.4% sensitivity (Fig. 1C).

## Discussion

Our cross‐sectional study provides compelling evidence that balance impairment is an early feature of HD and aligns with earlier research, suggesting that postural instability might emerge before overt HD symptoms.^8^, ^12^, ^24^, ^25^, ^26^, ^27^, ^28^ By across the HD‐ISS spectrum in our cross‐sectional sample, we expand on previous work by demonstrating that balance dysfunction is present early in the disease course and worsens with subsequent HD stage. Although preliminary, the increased TBS in stage 0/1 participants, compared to HCs, raises the possibility that subtle balance deficits may begin during early caudate and putamen neurodegeneration, prior to the onset of overt HD symptoms.^29^ This finding is supported by evidence from several animal studies: YAC128 and CAG140 knock‐in mice demonstrate postural abnormalities before the appearance of overt motor symptoms.^26^, ^27^, ^30^ In addition, early degeneration of both caudate and putamen likely contributes to balance dysfunction through distinct mechanisms: putamen involvement affects automatic postural responses and muscle tone regulation, whereas caudate degeneration compromises the cognitive–motor integration required for postural stability.^2^, ^3^ However, there remains a need to directly correlate these functional deficits with concurrent structural brain changes to better understand the neural mechanisms underlying early balance impairment.

The progressive nature of balance dysfunction in HD becomes clear with our observation of a steady increase in TBS from stage 0/1 (9.56 cm) to stage 2 (14.46 cm) to Stage 3 (28.26 cm). This progression aligns with concurrent declines in cognitive function and escalation of motor impairment, suggesting that balance impairment represents 1 facet of a broader pattern of neurological decline.^16^ Particularly noteworthy is the marked distinction between stages 2 and 3, where we identified a threshold of 11.85 cm with 82.4% sensitivity. This finding suggests that TBS measurements could serve as a valuable indicator of transition to functional decline, offering clinicians a concrete metric for identifying patients who may need additional intervention.

Our study addresses the limitations of previous research by utilizing the HD‐ISS staging system, which allows for a more precise characterization of balance impairment across the disease spectrum compared to traditional methods. The use of the PIN score as a proxy for HD‐ISS staging provides a valuable alternative in the absence of volumetric magnetic resonance imaging data, enabling wider applicability of this staging system in research and clinical settings.

Our findings provide preliminary evidence that incorporation of objective balance assessments could be worthwhile in future clinical trials. The early detection of balance impairment in stage 0/1 participants and identification of clinically relevant thresholds support the potential value of postural stability measures as endpoints for HD clinical research. The emergence of portable technologies such as inertial measurement units and wearable sensors may facilitate broader clinical implementation and remote monitoring in HD populations, potentially enhancing future clinical trials through continuous assessment of balance changes in early disease stages.^28^

However, our study is not without limitations. The cross‐sectional design precludes definitive conclusions about the longitudinal progression of balance impairment. Future longitudinal studies are needed to track changes in balance over time and confirm these findings. Incorporating other balance assessments, such as gait analysis, could provide a more comprehensive understanding of balance dysfunction in HD.

Despite these limitations, our findings have important implications for clinical practice and future research. Early detection of balance impairment can facilitate timely interventions, such as physical therapy and fall prevention strategies, to maintain functional independence and improve quality of life for individuals with HD. Furthermore, our results emphasize the need for incorporating balance assessment into routine clinical evaluations for HD, particularly in the early stages. This can aid in monitoring disease progression, identifying individuals at risk of functional decline, and guiding personalized management strategies.

In conclusion, this cross‐sectional study demonstrates that balance impairment is an early and progressive feature of HD, detectable even before the onset of overt motor symptoms. The use of the HD‐ISS and a brief balance assessment tool provides a valuable framework for characterizing balance dysfunction across the disease spectrum. Our findings highlight the importance of early detection and management of balance impairment in HD to optimize functional capacity and improve quality of life for individuals living with this devastating disease.

## Author Roles

(1) Research project: A. Conception, B. Organization, C. Execution; (2) Statistical analysis: A. Design, B. Execution, C. Review and critique; (3) Manuscript preparation: A. Writing of the first draft, B. Review and critique.

J.K.: 1A, 1B, 1C, 2A, 2B, 2C, 3A

N.Y.: 1B, 1C, 2C, 3B

K.B.: 1B,1C, 2C, 3B

A.H.: 1B, 1C, 3B

P.E.G.: 1A, 2C, 3B

D.J.G.: 1A, 2C, 3B

J.C.‐B.: 1A, 2B, 2C, 3A, 3B

## Disclosures

**Ethical Compliance Statement:** Institutional Review Board approval was obtained from the Institutional Review Board of the University of California, San Diego (protocol no. 170038). Written informed consent was obtained from all participants before participation. We confirm that we have read the journal's position on issues involved in ethical publication and affirm that this work is consistent with those guidelines.

**Funding Sources and Conflicts of Interest:** No specific funding was received for this work. The authors declare that there are no conflicts of interest relevant to this work.

**Financial Disclosures for the Previous 12 Months:** D.J.G. is eligible for royalties from a patent (US Patent 10,660,558, 2020) related to BTrackS Balance Plate. In addition, he has an equity stake (stock options) in Balance Tracking Systems, Inc. This financial conflict of interest is mitigated by a management plan put in place by his academic institution to ensure the integrity of his research.

## Data Availability

The data that support the findings of this study are available from the corresponding author upon reasonable request.
